# Alteration of contrast enhanced ultrasound (CEUS) of hepatocellular carcinoma in patients with cirrhosis and transjugular intrahepatic portosystemic shunt (TIPS)

**DOI:** 10.1038/s41598-020-77801-9

**Published:** 2020-11-26

**Authors:** Johannes Chang, Alexia Dumitrache, Nina Böhling, Jasmin Abu-Omar, Carsten Meyer, Deike Strobel, Julian Luetkens, Andreas Minh Luu, Jürgen Rockstroh, Christian P. Strassburg, Jonel Trebicka, Maria A. Gonzalez-Carmona, Milka Marinova, Michael Praktiknjo

**Affiliations:** 1 Department of Internal Medicine I, University Hospital Bonn, Venusberg-Campus 1, 53127 Bonn, Germany; 2 Department of RadioIogy, University Hospital Bonn, Bonn, Germany; 3 Department of Internal Medicine I, University Hospital Erlangen-Nuremberg, Erlangen, Germany; 4 Department of General and Visceral Surgery, St. Josef Hospital, University of Bochum, Bochum, Germany; 5 Department of Internal Medicine I, University Hospital Frankfurt, Frankfurt, Germany; 6 European Foundation for the Study of Chronic Liver Failure, Barcelona, Spain

**Keywords:** Liver cancer, Liver cirrhosis, Portal hypertension

## Abstract

Transjugular intrahepatic portosystemic shunt (TIPS) can treat portal hypertensive complications and modifies hepatic hemodynamics. Modification of liver perfusion can alter contrast enhancement dynamics of liver nodules. This study investigated the diagnostic performance of contrast-enhanced ultrasound (CEUS) to diagnose hepatocellular carcinoma (HCC) in cirrhosis with TIPS. In this prospective monocentric observational study, CEUS was used to characterize focal liver lesions in patients at risk for HCC with and without TIPS. Times of arterial phase hyperenhancement (APHE) und washout were quantified. Perfusion-index (PI) and resistance-index (RI) of hepatic artery and portal venous flow parameters were measured via doppler ultrasonography. Diagnostic gold standard was MRI/CT or histology. This study included 49 liver lesions [23 TIPS (11 HCC), 26 no TIPS (15 HCC)]. 26 were diagnosed as HCC by gold standard. Sensitivity and specificity of CEUS to diagnose HCC with and without TIPS were 93.3% and 100% vs. 90.9% and 93.3%, respectively. APHE appeared significantly earlier in patients with TIPS compared to patients without TIPS. TIPS significantly accentuates APHE of HCC in CEUS. CEUS has good diagnostic performance for diagnosis of HCC in patients with TIPS.

## Introduction

Advanced chronic liver disease is a growing health care burden worldwide^1,2^. Complications such as refractory ascites and variceal bleeding contribute to morbidity and mortality of these patients^3^. Another severe complication increasing its mortality is the development of hepatocellular carcinoma (HCC) in cirrhotic livers. HCC ranks fifth in frequency worldwide and prognosis strongly depends on stage at diagnosis^4^. Therefore, early diagnosis of HCC is crucial and HCC screening via B-mode ultrasound is recommended^2,5,6^. Interestingly, unlike other carcinoma, the diagnosis of HCC across national and international guidelines does not require histologic confirmation, if contrast enhanced imaging techniques show typical contrast hyperenhancement in arterial phase and mild washout in venous phase^4,7,8^. Among computed tomography (CT) and magnetic resonance imaging (MRI), contrast-enhanced ultrasound (CEUS) can be used to diagnose HCC lesions. CEUS has shown high accuracy in diagnosis of HCC as reported in various multicentric trials^9–11^. This led to incorporation of CEUS in the diagnostic algorithm of some current guidelines^4,6,12^. CEUS has a higher temporal resolution compared to CT or MRI and might therefore be more sensitive in identifying phase specific differences in contrast enhancement^13,14^.

The diagnostic algorithm for HCC in high risk patients is widely performed according to Liver Imaging Reporting and Data System (LI-RADS)^8^. This algorithm accounts for size, arterial phase hyperenhancement and wash out phenomenon in later phases. Arterial phase hyperenhancement (APHE) of liver lesions is a major determining factor for the classification of HCC by CEUS^9,11,15^*.* APHE reflects the process of arterial angiogenesis which is a key component of HCC pathogenesis, where predominantly arterial rather than portal-venous blood supply is drawn by HCC^16^.

HCC occurs particularly among high risk patients such as patients with liver cirrhosis of any cause and chronic viral hepatitis^2,6^. In selected patients with decompensated liver cirrhosis, implantation of TIPS can improve survival^3,17,18^. TIPS decompresses the portal-venous system by redirecting portal venous blood flow from the intrahepatic portal-venous branches through the TIPS tract and thus resulting in a decreased portal venous blood flow to the liver. This triggers a compensatory mechanism known as the hepatic arterial buffer response, which increases hepatic arterial blood flow to the liver^19–21^. Thereby, the liver perfusion is modified and dynamics of contrast enhancement are subsequently altered^20,22^. This suggests altered perfusion of focal liver lesions and therefore their characteristics of contrast enhancement. However, contrast enhancement of focal liver lesions in CEUS in patients with TIPS has not been studied yet. This monocentric observational study investigated CEUS as a diagnostic tool and evaluated its diagnostic performance for the detection of focal liver lesions suspicious of HCC in patients with cirrhosis with and without TIPS.

## Methods

### Patient cohort

This is an analysis including focal liver lesions of cirrhotic patients with and without TIPS from the prospective NICETIES cohort from our tertiary center (clinicaltrials.gov identifier: NCT03746210). CEUS was used to characterize focal liver lesions that were identified during HCC screening via B-mode ultrasound. Liver lesions were then confirmed by at least one more contrast-enhanced imaging (CT and/or MRI). Diagnostic gold standard was MRI/CT or histology. The choice of CT vs. MRI was made according to the physician’s discretion and mostly depended on the patient’s renal function and history of radiation exposure. For MRI, a commercially available clinical 3.0 T MR imaging system (Ingenia 3.0 T; Philips Healthcare, Best, Netherlands) or a 1.5 T MR imaging system (Ingenia 1.5 T; Philips Healthcare, Best, Netherlands) and for CT commercially available clinical CT imaging systems (Philips Brilliance 64 or Philips Brilliance 256 iCT, both Philips Healthcare, Best, The Netherlands) were used. For diagnosis of focal liver lesions standardized imaging protocols for MRI and CT were used according to our institution’s standards. All imaging data were reviewed and interpreted by the same experienced radiologist. Histology slides were assessed by the same pathologist. This study was approved by the ethics committee of the University of Bonn, Faculty of Medicine (number 133/18) and performed in accordance to the declaration of Helsinki. Informed written consent has been obtained from all participants.

### Contrast enhanced ultrasound (CEUS)

Contrast enhanced ultrasound (CEUS) was used to examine liver lesions in patients at risk of HCC (liver cirrhosis). Baseline sonography and CEUS were performed using the same ultrasound scanner (Supersonic Aixplorer, Supersonic Imaging). All patients were investigated after a 6-h fasting period. First, the liver was examined with baseline ultrasound to locate focal liver lesions. A sulphur hexafluoride-filled microbubble contrast agent, SonoVue (Fa. Bracco, Italy) was injected into a peripheral vein in a common manner. The dynamics of the contrast enhancement were observed for at least 5 min. The examination was performed by an experienced DEGUM (German Society of Ultrasound in Medicine) certified specialist in Internal Medicine.

Arterial phase hyperenhancement (APHE) and washout of contrast medium was quantified. A recent multicenter study showed that 90–99% nodules classified as CEUS LI-RADS 4 and 5 were HCC^9^. Moreover, APHE is key feature for the correct diagnosis of HCC in CEUS^23^. Therefore, in this study focal liver lesions classified as CEUS LI-RADS-4 and -5 were considered HCC in this study.

### Hepatic perfusion parameters

Perfusion-index (PI) and resistance-index (RI) of hepatic artery and portal venous flow parameters were measured via doppler ultrasonography.

### Statistical analysis

Data analysis was performed using SPSS (version 25, IBM, Armonk, NY, USA). For all variables descriptive statistics were computed. Non-parametric testing was used to compare the data of both groups. Continuous variables are expressed as median (range). Categorical variables are presented as absolute cases or percentage. The sensitivity, specificity, positive predictive value (PPV) and negative predictive value (NPV) were analyzed. A two-tailed p-value < 0.05 was considered statistically significant.

## Results

### Patient characteristics

In total, 49 focal liver lesions from 33 patients with liver cirrhosis were included in this study (median age 61 years, 16 male). 18 patients had TIPS. Median MELD and Child–Pugh score were 13 (6–24) and 7 (5–11) respectively. Etiology of liver cirrhosis was mostly referred to alcohol in 25 (76%) patients. The indication for TIPS was refractory ascites in 12 (67%) and variceal bleeding in 6 (33%) patients. AFP was not significantly different between both groups (Table 1). There was no significant difference in the distribution of patients with solitary/multiple lesions, the number of patients with HCC, the number of lesions and number of HCC lesions between the TIPS and no-TIPS groups.

**Table 1:** General patient characteristics.

	Parameter	all	TIPS	no TIPS
		n = 33	n = 18	n = 15
Baseline general	Age (years)	61 (33–81)	58 (33–79)	64 (38–81)
	Sex male/female	16/17 (48.5%/51.5%)	8/10 (44,4%/55.6%)	8/7 (53.3%/46.7%)
	Etiology of cirrhosis alcohol/viral/others	25/1/7 (76%/3%/21%)	14/0/4 (78%/0%/22%)	11/1/3 (73%/7%/20%)
	TIPS Indication (ascites/variceal bleeding)		12/6 (67%/33%)	
Baseline clinical events	PVT	4 (12%)	3 (17%)	1 (7%)
	Ascites	18 (54.5%)	15 (84.4)*	3 (20)
	Hepatic Encephalopathy	8 (24%)	3 (17%)	5 (33%)
Baseline score	MELD	13 (6–24)	12 (9–18)	13 (6–24)
	Child–Pugh score	7 (5–11)	6 (5–9)	8 (5–11)**
	Child–Pugh class A/B/C	12/18/3 (36%/55%/9%)	10/8/0 (56%/44%/0%)	2/10/3** (13%/67%/20%)
Baseline laboratory	Creatinine [mg/dl]	0.8 (0.6–2.1)	0.9 (0.6–3.5)	0.9 (0.6–2.1)
	Bilirubin [mg/dl]	1.7 (0.2–7.6)	1.7 (0.6–3.4)	1.4 (0.2–7.6)
	AST [U/l]	43 (20–367)	43 (20–136)	47 (20–367)
	ALT [U/l]	36 (14–127)	36 (17–71)	30 (14–127)
	Albumin [g/l]	33 (2–47)	34 (14–47)	27 (2–45)**
	AFP [ng/ml]	3.9 (1.8–797)	3.25 (1.8—124.3)	10.4 (1.8—797)
	INR	1.2 (1–2)	1.3 (1–2)	1.1 (1–1.7)
	Platelets [× 10^9^/L]	147 (46–451)	138 (54–241)	158 (46–451)
HCC/FLL	Solitary/multiple lesions	24/9 (73/27%)	14/4 (78/22%)	10/5 (67/33%)
	Number of lesions per patient	1 (1–8)	2 (1–8)	1 (1–6)
	Number of HCC lesions per patient	1 (1–4)	1 (1–3)	1 (1–4)
	Distribution of HCC (HCC/no HCC)	21/12 (64/36%)	11/7 (61/39%)	10/5 (67/33%)

*TIPS* transjugular intrahepatic portosystemic shunt, *PVT* portal vein thrombosis, *MELD* model of end-stage liver disease, *AST* aspartate aminotransferase, *ALT* alanine aminotransaminase, *AFP* alpha-Fetoprotein, *INR* International Normalized Ratio, *FLL* focal liver lesions, *HCC* hepatocellular carcinoma, p-value: *p < 0.05, **p < 0.01, ***p < 0.001.

### Characteristics of liver lesions

Median diameter of liver lesions was similar between TIPS and non-TIPS patients (2.3 cm (0.7–4.6 cm) vs. 3.2 cm (0.9–15.3 cm)). There was one outlier in the non-TIPS group (15.3 cm), without this outlier the median diameter was 2.5 cm (0.9–5.7 cm). Median depth of the liver nodules from skin was not significantly different between the two groups either [TIPS: 7 cm (3–14 cm) vs. no-TIPS: 5 cm (2–11 cm)] (Table 2). Contrast-enhanced MRI, CT and histology were available as reference in 37 (76%) (n = 21 (81%) with TIPS), 38 (78%) (n = 19 (73%) with TIPS) and 10 (20%) (n = 3 (12%) with TIPS) patients, respectively (Table 2). Twelve (25%) (n = 5 (19%) with TIPS) had only CT, 11 (22%) (n = 7 (27%) with TIPS) had only MRI, and 26 (53%) (n = 14 (54%) with TIPS) had both, MRI and CT (Table 2). All biopsies in the TIPS group were taken during local ablative therapy (n = 3, 100%). In the group without TIPS biopsies were obtained either during surgery or done due to inconclusive imaging (n = 3 (43%) and n = 4 (57%) respectively). (Supplementary Table 2A).

**Table 2:** Characteristics of liver lesions, n = 49, (with number, diameter of liver lesions depth of liver nodules from skin in cm), diagnostic gold standard (only CT/only MRI/ CT and MRI), duplexsonography data showing perfusion-index (PI) and resistance-index (RI) hepatic artery and portal venous flow parameters, and diagnosis of liver lesions according to diagnostic gold standard (histology/CT/MRT).

	Parameter	All (n = 49)	TIPS (n = 26)	No TIPS (n = 23)
Characteristics of liver lesions	Number of liver lesions per patient	2 (1–8)	2 (1–8)	2 (1–6)
	Depth of lesion from skin [cm]	6 (2–14)	7 (3–14)	5 (2–11)
	Diameter of lesion [cm]	2.3 (0.7–15.3)	2.3 (0.7–4.6)	3.2 (0.9–15.3)
Diagnostic gold standard	MRI	37 (76%)	21 (81%)	16 (70%)
	CT	38 (78%)	19 (73%)	19 (82%)
	only CT/only MRI/CT and MRI	12/11/26 (25/22/53%)	5/7/14 (19/27/54%)	7/4/12 (30/18/52%)
	Histology	10 (20%)	3 (12%)	7 (30%)
Duplex sonography	PVV (cm/s)	28 (0–88)	35 (22–88)	21 (0–37)***
	Hepatic artery RI	0.8 (0.6–0.9)	0.8 (0.6–0.9)	0.7 (0.6–0.9)
	Hepatic artery PI	1.6 (0.8–3.3)	1.6 (1.1–3.3)	1.6 (0.8–2.7)
Diagnosis	HCC	26 (53%)	11 (42%)	15 (65%)
	Benign lesions	23 (47%)	15 (58%)	8 (40%)

*TIPS* transjugular intrahepatic portosystemic shunt, *MRI* magnetic resonance imaging, *CT* computed tomography, *PVV* portal vein velocity, *RI* Resistance-index, *PI* Perfusion-index, *HCC* hepatocellular carcinoma, p-value: *p < 0.05, **p < 0.01, ***p < 0.001.

The examination of liver lesions by the gold standard resulted in 26 HCC (53%), 11 (42%) of them in patients with TIPS. 23 liver lesions (47%) were regenerative nodules or other benign lesions (Supplementary Table 4). There was no significant difference in distribution of HCC between TIPS and no-TIPS group (Table 2).

### Characteristics of biopsied lesions

Of the 10 lesions biopsied, 7 (70%) were HCC. HCCs were graded into 4 G1 (57%), 1 G2 (14%) and 2 G3 (29%) respectively. All 7 (100%) of the biopsied HCC lesions showed APHE, 5 (71%) showed washout. The 2 lesions that did not show washout were G1 HCC. Six (86%) of the HCC were correctly classified identically by CT/MRI and CEUS, respectively (Supplementary Table 3). Reason for biopsy of the benign lesions (3 (30%)) were inconclusive imaging and showed one abscess and two regenerative nodules. The benign lesions had neither shown APHE nor washout. Clinical characteristics of biopsied patients are shown in Supplementary Table 2B.

### Hepatic hemodynamics

Median portal vein velocity (PVV) was 32 cm/s (22–88 cm/s) vs. 21 cm/s (0–37 cm/s) (p < 0.001) in patients with and without TIPS, respectively. Perfusion-index (PI) of hepatic artery similar between the two groups [TIPS: 1.6 (1.1–3.3) vs. no-TIPS 1.6 (0.8–2.7)]. Resistance-index (RI) of hepatic artery was higher in TIPS patients [0.8 in (0.6–0.9)] compared to no-TIPS patients [0.7 (0.6–0.9)] (Table 2).

### Diagnostic performance of CEUS

53% (n = 26) of the focal liver lesions were classified as CEUS LI-RADS-4 or LI-RADS-5. 53% (n = 26) of the liver lesions showed APHE, 65% (n = 15) of them in patients with TIPS (Supplementary Table 1). A washout in venous phase after at least 120 s was detectable in 43% (n = 21) of the liver lesions, 22% (n = 11) of them in patients with TIPS (Table 3). 10% (n = 5) did show APHE but no washout.

**Table 3:** Characteristics of contrast enhancement of focal liver lesions (n = 49).

Characteristics of contrast enhancement	All (n = 49)	TIPS (n = 23)	No TIPS (n = 26)
APHE	26 (53%)	15 (65%)	11 (42%)
washout in venous phase	21 (43%)	11 (22%)	10 (20%)

*APHE* arterial phase hyperenhancement.

The diagnostic performance of CEUS was analyzed with the gold standard as reference. Sensitivity, specificity, positive predictive value, negative predictive value and diagnostic value to diagnose HCC in TIPS patients was 93.3%, 100%, 100%, 88.9%, 96% and in non-TIPS patients 90.9%, 93.3%, 90.9%, 93.3%, 92% (Table 4). The rate of correctly classified HCC was similar in both groups, 14 of 15 (93%) lesions and 10 out of 11 (91%) in the TIPS and the no-TIPS group, respectively.

**Table 4:** Classification of HCC diagnosis by CEUS according to final diagnosis.

	CEUS					
	All (n = 49)		TIPS (n = 26)		No TIPS (n = 23)	
	HCC	Benign	HCC	Benign	HCC	Benign
HCC	24 (49%)	1 (2%)	10 (38%)	1 (4%)	14 (61%)	0 (0%)
Benign	2 (4%)	22 (44%)	0 (4%)	15 (58%)	1 (4%)	8 (35%)

*HCC* Hepatocellular carcinoma, *TIPS* transjugular intrahepatic portosystemic shunt, *CEUS* contrast-enhanced ultrasound.

### Quantitative assessment of contrast enhancement

Quantitative analysis of APHE timing showed eight seconds earlier APHE in patients with TIPS compared to non-TIPS patients, which was statistically significant (Table 5). The start of venous washout time and time to be washed out completely was later in the TIPS group. Moreover, there was a trend towards higher rates of lesions without washout in non-TIPS patients (Table 5). CEUS of all benign lesions, except for 3 referred to biopsy, showed characteristic patterns for the respective lesions (regenerative nodule, hemangioma, abscess).

**Table 5:** Measurement of APHE and Washout. Comparison of APHE- and venous washout onset (in seconds) of HCC lesions (n = 26).

Measurement of APHE and washout	All (n = 26)	TIPS (n = 11)	No TIPS (n = 15)
APHE start (s)	14 (4–21)	9 (4–12)***	17 (13–21)
Washout start (s)	140 (33–259)	140 (33–242)	126 (124–259)
Washout (before 180 s/after 180 s/no washout)	16/5/5 (61/19/19%)	9/2/0 (82/18/0%)	7/3/5 (47/20/33%)
Washout end (s)	246 (87–303)	247 (87–260)	186 (185–303)

*APHE* arterial phase hyperenhancement, *HCC* hepatocellular carcinoma, p-value: *p < 0.05, **p < 0.01, ***p < 0.001.

## Discussion

This study is the first to highlight the significantly altered APHE in CEUS of HCC in patients with TIPS (Supplementary Figure 1). Moreover, it confirms the diagnostic performance of CEUS for HCC and expands it to patients with TIPS.

It is of special importance, because TIPS is indicated in selected patients with decompensated liver cirrhosis^24^, which are at high risk of developing HCC^4^. HCC is one of the few malignancies with an increasing mortality rate. Early diagnosis is crucial for prognosis. Therefore, good diagnostic and easily accessible tools in patients with TIPS that distinguish between malignant and benign lesions are an unmet need. Current international guidelines recommend regular HCC screening twice a year via B-mode ultrasound^25^. However, due to conflicting reports of low specificity, CEUS is debated across national and international guidelines^4,7,8,26^. Nonetheless, in clinical practice some patients have contraindications for contrast media and CT/MRI scans are not widely available in all health care systems. In these situations, CEUS plays an important role as diagnostic tool. In this study, we confirm the good diagnostic performance of CEUS for HCC in cirrhosis, underlining the robustness of our data^9,10^. Moreover, we expand the use of CEUS to patients with TIPS without significant change in diagnostic performance, suggesting routine use in these patients.

This is not necessarily expected because diagnostic algorithms such as CEUS LI-RADS rely on APHE and washout in later phases. Concerning liver perfusion, TIPS severely reduces portal venous perfusion of the liver, resulting in a compensatory increase of hepatic arterial perfusion^17,19^. This could lead to the assumption that perfusion of liver focal liver lesions in patients with TIPS might be altered from “regular” perfusion. Indeed, HCC perfusion mainly relies on arterial neovascularization, which is reflected in CEUS LI-RADS criteria and even more emphasized in other algorithms^23^. According to our data, APHE is not only maintained but rather significantly accentuated after TIPS, making it possible for HCC detection with CEUS in patients with TIPS. However, CEUS performers need to be aware of the earlier timing of APHE in these patients.

An important issue for the treatment and prognosis is the distinction of HCC from other malignancies, cholangiocellular carcinoma in particular. Conflicting data led to degrading of CEUS in guidelines in the past^2^. Marked washout of the contrast agent in the portal venous phase or late phase is a common feature of most metastatic lesions and cholangiocellular carcinoma, while APHE patterns can differ between different kinds of malignant lesions. The washout pattern of HCC is typically mild, gradual and occurs in later phases. In intrahepatic cholangiocellular carcinoma and other malignancies, the washout appears earlier and more pronounced^27^. Venous washout of HCC begins later and requires a longer time to be washed out completely in our patients with TIPS compared to those without. Nevertheless, median start of washout was in the late phase after 120 s. The longer period of washout is in line with previous observations with rather mild (not fast and marked) washout in HCC lesions. The trend of longer washout in the TIPS group vs. no-TIPS group might also be due to the changed hepatic hemodynamics and –perfusion after TIPS placement. However, more studies with more detailed quantification of venous washout, i.e. via computerized time intensity curve analysis are needed to confirm our data.

Of note, in our study, two biopsied HCC that did not show washout were G1 HCC. In these cases in/correct classification by CT/MRI and CEUS were identical, suggesting the diagnostic performance to be associated to tumor grading. More studies including more patients are needed to investigate whether tumor grading and subclassification of HCC show different enhancement patterns.

In both of our study cohorts with and without TIPS, the non-HCC lesions showed characteristic contrast-enhancement patterns, suggesting no significant impact of TIPS in those lesions. However, larger studies to characterize non-HCC liver lesions in patients with TIPS and their differentiation are needed as well to confirm our results.

To date, routine HCC screening is done by B-mode ultrasound. Though CEUS has been successfully described in the context of surveillance after HCC treatment^28–31^, data regarding the use of CEUS for routine HCC screening is at best scarce^32,33^. Currently, CEUS is recommended as a good diagnostic tool for the work up of focal liver lesions found in routine HCC screening via B-mode. Our data support the good diagnostic accuracy for HCC and expands it to patients with TIPS, who often are at risk for HCC development.

There are several limitations to this study, it is a monocentric study and the sample size is relatively small. However, despite the small sample size, this study is the first, aiming to establish CEUS as an accurate diagnostic tool for characterizing HCC in patients with TIPS. This should stimulate future prospective multicenter studies with larger cohorts for validation. Moreover, the gold standard would ideally be histology in all focal liver lesions. However, biopsy is not the standard of diagnostic for HCC according to guidelines. Interestingly, in this cohort MRI was performed more frequently in the TIPS group, which can be attributed to the higher rate of renal dysfunction being a contraindication for CT contrast medium in those patients.

In conclusion, this study shows first data of CEUS as an accurate diagnostic tool for characterizing HCC lesions in patients with TIPS. It shows an alteration of the APHE, and possibly venous washout pattern compared to patients without TIPS.

## Supplementary information

Supplementary Information 1.

Supplementary Information 2.

## Abbreviations

CEUS: Contrast-enhanced ultrasound
TIPS: Transjugular intrahepatic portosystemic shunt
PVT: Portal vein thrombosis
MELD: Model of end-stage liver disease
CLIF-C-AD: CLIF consortium acute decompensation score
CLIF-C-ACLF: CLIF consortium acute-on-chronic liver failure
WBC: White-blood-cells
MRI: Magnetic resonance imaging
CT: Computed tomography
PVV: Portal vein velocity
RI: Resistance-index
PI: Perfusion-index
HCC: Hepatocellular carcinoma
APHE: Arterial phase hyperenhancement
LI-RADS: Liver Imaging Reporting and Data System
