# Supplementary material for: Alteration of contrast enhanced ultrasound (CEUS) of hepatocellular carcinoma in patients with cirrhosis and transjugular intrahepatic portosystemic shunt (TIPS)
Source: Sci Rep. 2020 Nov 26;10:20682. doi: 10.1038/s41598-020-77801-9 (PMC7692482; doi:10.1038/s41598-020-77801-9)
Supplement: Supplementary file 1 — Supplementary Information 1. [file 41598_2020_77801_MOESM1_ESM.pdf]

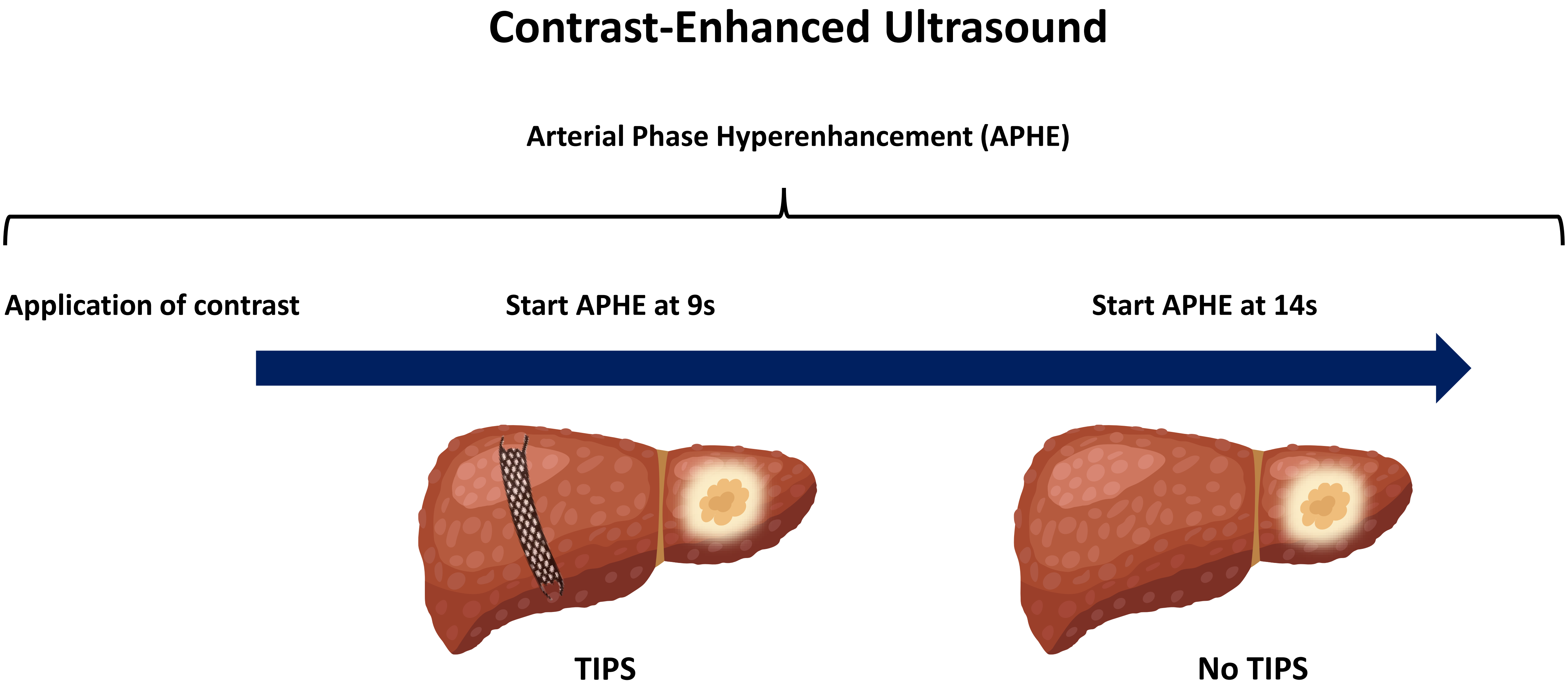
Contrast-Enhanced Ultrasound
Arterial Phase Hyperenhancement (APHE)
Application of contrast
Start APHE at 9s
Start APHE at 14s
TIPS
No TIPS
