# Supplementary material for: Alteration of contrast enhanced ultrasound (CEUS) of hepatocellular carcinoma in patients with cirrhosis and transjugular intrahepatic portosystemic shunt (TIPS)
Source: Sci Rep. 2020 Nov 26;10:20682. doi: 10.1038/s41598-020-77801-9 (PMC7692482; doi:10.1038/s41598-020-77801-9)
Supplement: Supplementary file 2 — Supplementary Information 2. [file 41598_2020_77801_MOESM2_ESM.docx]

**Alteration of contrast enhanced ultrasound (CEUS) of hepatocellular carcinoma in patients with cirrhosis and transjugular intrahepatic portosystemic shunt (TIPS)**

Johannes Chang^1*^, Alexia Dumitrache^1*^, Nina Böhling^1*^, Jasmin Abu-Omar^1^, Carsten Meyer^2^, Deike Strobel^3^, Julian Luetkens^2^, Andreas Minh Luu^4^, Jürgen Rockstroh^1^, Christian P. Strassburg^1^, Jonel Trebicka^5,6^, Maria A. Gonzalez-Carmona^1^, Milka Marinova^2^, Michael Praktiknjo^1+^

^1^ Department of Internal Medicine I, University Hospital Bonn, Germany

^2^ Department of RadioIogy, University Hospital Bonn, Germany

^3^ Department of Internal Medicine I, University Hospital Erlangen-Nuremberg, Germany

^4^ Department of General and Visceral Surgery, St. Josef Hospital, University of Bochum, Germany

^5^ Department of Internal Medicine I, University Hospital Frankfurt, Germany

^6^ European Foundation for the Study of Chronic Liver Failure, Barcelona, Spain

*** contributed equally as first author**

**^+^ corresponding author**

**Short title: TIPS alters CEUS pattern of HCC**

**Corresponding author:** Dr. med**.** Michael Praktiknjo

Department of Internal Medicine I, University of Bonn, Venusberg-Campus 1, 53127 Bonn. Email: [Michael.praktiknjo@ukbonn.de](mailto:Michael.praktiknjo@ukbonn.de), Tel. +49 228 287 15259

**Supplementary Table 1:**

**LI-RADS classification.** Distribution of focal liver lesions in categories 1-5 according to the LI-RADS classification.

| **CEUS LI-RADS** | **1** | **2** | **3** | **4** | **5** |
| --- | --- | --- | --- | --- | --- |
| all (n=49) | 13 (27%) | 3 (6%) | 7 (14%) | 7 (14%) | 19 (39%) |
| TIPS (n=23) | 4 (17%) | 0 (0%) | 4 (17%) | 5 (22%) | 10 (44%) |
| no TIPS (n=26) | 9 (35%) | 3 (12%) | 3 (12%) | 2 (8%) | 9 (35%) |

Abbreviations: LI-RADS (Liver Imaging Reporting and Data System), TIPS (transjugular intrahepatic portosystemic shunt), HCC (hepatocellular carcinoma)

**Supplementary Table 2 A:**

**Histology.** Percentage and reason for histology of HCC lesions as reference standard.

| **reason for biopsy** | **all (n=10)** | **TIPS (n=3)** | **no TIPS (n=7)** |
| --- | --- | --- | --- |
| during operation / inconclusive imaging / at local ablative therapy | 3/4/3 (30/40/30%) | 0/0/3 (0/0/100%) | 3/4/0 (43/57/0%) |

Abbreviations: TIPS (transjugular intrahepatic portosystemic shunt)

**Supplementary Table 2 B:**

**Characteristics of biopsied patients.**

| **Parameter** | **all (n = 10)** | **TIPS (n=3)** | **no TIPS (n=7)** |
| --- | --- | --- | --- |
| Sex (male / female) | 6/4 (60/40%) | 2/1 (67/33%) | 4/3 (57/43%) |
| Age | 60 (39-77) | 57 (56-71) | 63 (39-77) |
| Ätiologie (alcohol/HBV) | 8/2 (80/20%) | 3/0 (100%) | 5/2 (79/21%) |
| MELD | 9 (9-16) | 12 (9-13) | 9 (6-19) |
| Child-Score | 7 (5-9) | 6 (5-8) | 7 (5-9) |
| Child-Class (A/B) | 4/6 (40/60%) | 2/1 (67/33%) | 2/5 (29/71%) |

Abbreviations: HBV (hepatitis B Virus), MELD (Model for End Stage Liver Disease), TIPS (transjugular intrahepatic portosystemic shunt)

**Supplementary Table 3:**

Incidence of APHE and washouts according to tumor grading. Correctly classified tumors in CT/MRI and CEUS according to tumor grading.

|  |  | **HCC grading** | | |
| --- | --- | --- | --- | --- |
|  |  | **G1** | **G2** | **G3** |
| **all (n = 7)** | 7 (100%) | 4 (57%) | 1 (14%) | 2 (29%) |
| **aPHE** | 7 (100%) | 4 (57%) | 1 (14%) | 2 (29%) |
| **washout** | 5 (71%) | 2 (29%) | 1 (14%) | 2 (29%) |
|  |  |  |  |  |
| **lesions correctly classified:** |  |  |  |  |
| **CT/MRI** | 6 (86%) | 4 (57%) | 1 (14%) | 1 (14%) |
| **CEUS** | 6 (86%) | 4 (57%) | 1 (14%) | 1 (14%) |

Abbreviations: HCC (hepatoceulluar carcinoma), aPHE (arterial phase hyperenhancement, CT (computed tomography), MRI (magnetic resonance imaging, CEUS (contrast-enhanced ultrasound)

**Supplementary Table 4:**

List of benign liver lesions.

| **Benign lesions (n = 23)** | **n (%)** |
| --- | --- |
| Hemangioma | 4 (17%) |
| Regenerative nodule | 17 (74%) |
| Abscess | 2 (9%) |

**Supplementary Table 5:**

Classification of HCC diagnosis by CT/MRI according to final diagnosis.

|  | **CT** | | | | | |
| --- | --- | --- | --- | --- | --- | --- |
|  | **all (n=38)^+^** | | **TIPS (n=19)^++^** | | **no TIPS (n=19)^+++^** | |
|  | **HCC** | **benign** | **HCC** | **benign** | **HCC** | **benign** |
| **HCC** | 16 (42%) | 2 (5%) | 5 (26%) | 1 (5%) | 11 (58%) | 1 (5%) |
| **benign** | 0 (0%) | 15 (40%) | 0 (0%) | 10 (53%) | 0 (0%) | 5 (26%) |
|  | + 5 (13%), ++ 3 (16%), +++ 2 (11%) lesions of interest were not detected in this modality | | | | |  |
|  |  |  |  |  |  |  |
|  | **MRI** | | | | | |
|  | **all (n=37)** | | **TIPS (n=21)** | | **no TIPS (n=16)** | |
|  | **HCC** | **benign** | **HCC** | **benign** | **HCC** | **benign** |
| **HCC** | 18 (49%) | 0 (0%) | 8 (38%) | 0 (0%) | 10 (63%) | 0 (0%) |
| **benign** | 2 (5%) | 17 (46%) | 0 (0%) | 13 (62%) | 2 (12%) | 2 (25) |

Abbreviations: HCC (Hepatocellular carcinoma), TIPS (transjugular intrahepatic portosystemic shunt), CEUS (contrast-enhanced ultrasound), (CT (computed tomography), MRI (magnetic resonance imaging
